# Biomarkers during COVID-19: Mechanisms of Change and Implications for Patient Outcomes

**DOI:** 10.3390/diagnostics12020509

**Published:** 2022-02-16

**Authors:** Cheng-Han Chen, Sheng-Wen Lin, Ching-Fen Shen, Kai-Sheng Hsieh, Chao-Min Cheng

**Affiliations:** 1 Institute of Biomedical Engineering, National Tsing Hua University, Hsinchu 30013, Taiwan; gdc123.tw@gmail.com (C.-H.C.); wenwenlintw@gmail.com (S.-W.L.); 2 Department of Emergency Medicine, Taipei Veterans General Hospital, Taipei 11217, Taiwan; 3 School of Medicine, National Yang Ming Chiao Tung University, Taipei 11221, Taiwan; 4 Department of Pediatrics, National Cheng Kung University Hospital, College of Medicine, National Cheng Kung University, Tainan 70101, Taiwan; drshen1112@gmail.com; 5 Department of Pediatrics, Shuang Ho Hospital, Taipei Medical University, Taipei 23561, Taiwan

**Keywords:** COVID-19, SARS-CoV-2, biomarker, cytokine storm, point-of-care testing

## Abstract

As the COVID-19 (Coronavirus disease 19) pandemic spreads worldwide, the massive numbers of COVID-19 patients have created a considerable healthcare burden for every country. The clinical spectrum of SARS-CoV-2 infection is broad, ranging from asymptomatic to mild, moderate, severe, and critical. Most COVID-19 patients present with no or mild symptoms, but nearly one-fifth of all patients develop severe or life-threatening complications. In addition to localized respiratory manifestations, severe COVID-19 cases also show extra-pulmonary complications or induce multiorgan failure. Identifying, triaging, and treating patients at risk early is essential and urgent. This article reviews the potential prognostic value of various biomarkers at different clinical spectrum stages of COVID-19 infection and includes information on fundamental prognostic mechanisms as well as potential clinical implications. Biomarkers are measurable biochemical substances used to recognize and indicate disease severity or response to therapeutic interventions. The information they provide is objective and suitable for delivering healthcare providers with a means of stratifying disease state in COVID-19 patients. This, in turn, can be used to help select and guide intervention efforts as well as gauge the efficacy of therapeutic approaches. Here, we review a number of potential biomarkers that may be used to guide treatment, monitor treatment efficacy, and form individualized therapeutic guidance based on patient response. Implementation of the COVID-19 biomarkers discussed here may lead to significantly improved quality of care and patient outcomes for those infected with SARS-CoV-2 worldwide.

## 1. Introduction

COVID-19 (Coronavirus disease 19), caused by severe acute respiratory syndrome coronavirus 2 (SARS-CoV-2) infection, originated in Wuhan, China, in 2020 and quickly grew to pandemic levels, infecting approximately three hundred million people and claiming approximately five million lives worldwide [1]. This pandemic caused a tremendous socioeconomic impact due to necessary population isolation efforts including “lockdowns,” social distancing, and travel restrictions. The large numbers of COVID-19 patients also hoisted a considerable healthcare burden upon every country worldwide, stretching the capacities of limited human as well as material resources. Most COVID-19 patients present no symptoms or only mild, flu-like symptoms such as a dry cough or fever [2,3]. However, approximately 20% of all COVID-19 patients, develop severe or life-threatening complications, including hypoxia, respiratory failure, hemodynamic instability, and multiorgan failure [4]. For these severe cases, there were increased demands for intensive care efforts, mechanical ventilation equipment, and extracorporeal membrane oxygenation support [5]. The reported overall mortality rate for patients admitted to intensive care units (ICU) was approximately 30% [6], whereas the mortality rate for mildly afflicted COVID 19 patients was low [4]. Among those who survived severe infections, many subsequently suffer from long-term health issues [7].

In addition to localized respiratory manifestations, severe COVID-19 cases also present extra-pulmonary complications or multisystem inflammatory syndrome (MIS), which is more common in children [8]. During SARS-CoV-2 infection, both innate and adaptive host immune responses are triggered, increasing the potential for tissue and organ damage as a result of dysregulated host response and the body’s excessive pro-inflammatory reaction. The widespread and massive release of cytokines and chemokines, often called the “cytokine storm”, that ensues induces extrapulmonary involvement and possibly fatal complications associated with multisystem inflammatory syndrome [9,10]. It is, therefore, essential and urgent to identify, triage, and treat patients, especially those who may be progressing to severe disease stages, as early as possible.

Healthcare providers typically stratify COVID-19 patients based on clinical presentations, such as symptoms, peripheral pulse oxygen saturation, and blood pressure. These clinical assessments are essential but somewhat subjective, and sometimes only manifest at late stages of the disease. To identify at-risk patients early, some laboratory biomarkers can provide accessory, objective information that can significantly influence the quality of patient care.

Biomarkers are defined as measurable biochemical substances that are used to recognize and indicate the presence and severity of a disease, or a patient’s response to therapeutic interventions [11,12]. Various biomarkers are available to assess the severity of COVID-19 infection. These markers have various potential benefits: (1) identification and recognition of at-risk patients; (2) stratification of COVID-19 severity; (3) assistance in the establishment of admission or intensive care criteria; (4) treatment guidance via response assessment; (5) prognosis evaluation; and (6) ICU or ordinary ward discharge criteria framing [9]. These biomarkers can also be sorted into different categories: (1) host immune response markers that are immunological and inflammatory in nature; (2) hematological abnormality markers; and (3) end-organ injury and systemic response markers including, but not limited to, coagulation factors, cardiac enzymes, and renal function markers [9,13]. Please see Table 1 for biomarker examples associated with each of these categories.

## 2. Immunological and Inflammatory Response to SARS-CoV-2 Infection

As the SARS-CoV-2 virus enters host cells, binding to the angiotensin-converting enzyme 2 (ACE-2) receptor, viral replication in the cytoplasm releases many virions, causing local infection of neighboring cells and a viremia-induced systemic immune response [5]. Both innate and adaptive immune responses are therefore activated. Meanwhile, the SARS-CoV-2 also induces the secretion of soluble serum and urine ACE2, further releasing a vast quantity of cytokines and causing a systemic inflammatory response [14]. Excessive cytokines stimulate and recruit immune cells, causing dysregulated, overcompensating host responses leading toward “cytokine storm syndrome” [3,9]. This dysregulated immune response is a life-threatening, urgent-care condition that may be progressively characterized by persistent fever, nonspecific muscle pain, hemodynamic instability, disseminated intravascular coagulation (DIC), multiorgan failure, and, in the absence of suitable treatment, death [13].

### 2.1. Cytokines

The rapid deterioration of severely affected COVID patients may be attributable to an over-reacting immune system. This response may evolve from multiple pathways, including, but not limited to, NF-κB signaling, the JAK/STAT pathway, the NLRP3 pathway, and granulocyte macrophage-colony stimulating factor (GM-CSF) activation pathways [13,15]. While SARS-CoV-2 virus infects epithelial cells, the above immune pathways are activated, and several pro-inflammatory cytokines are released, including interleukin 1β, (IL-1β), IL-2, IL-6, IL-7, IL-8, IL-10, IL-17, TNF-α, and interferons (INFs) [3,9,13]. Elevated cytokine levels result in the recruitment of immune cells such as macrophages, T cells, and neutrophils to the infected area. Meanwhile, released serum cytokines such as interleukin-6 (IL-6), lead to increased synthesis of prototypic acute phase reactants, such as C-reactive protein (CRP), from the liver and into the bloodstream. Eventually, all of these dysregulated immune responses elicit various sequelae, such as the destabilization of endothelial cell-to-cell interactions, the destruction of the capillary and vascular barrier, tissue damage, and multiple organ failure, which may eventually result in the death of the infected individual [3,16].

The relationship between interleukin-6 and COVID-19 severity has been investigated by many studies [17,18,19]. Interleukin-6 (IL-6), a cytokine secreted by stimulated monocytes and macrophages, mediates a broad range of biological reactions [20]. Plasma IL-6 increases only 1 h after bodily insult and peaks after about 3–6 h [21,22]. Measurably increased IL-6 levels have been noted following trauma, stress, and infection [20]. A multifunctional cytokine that transmits cell signals and regulates immune cells, IL-6 has a strong proinflammatory effect associated with multiple biological functions and plays an important role in inflammation, tumor growth, and hematological diseases [23,24]. IL-6 triggers multiple immune responses by forming a positive feedback loop that eventually results in a cytokine storm, and elevated serum IL-6 may indicate disease severity and prognosis. Among COVID-19 patients admitted to the hospital, IL-6 was higher in the non-survival group than in the survival group [25,26]. Many studies have recognized IL-6 as a pivotal marker for implying the prognosis and severity of various diseases [27,28,29,30]. In addition, serum IL-6 may be useful for monitoring treatment response and evaluating the efficacy of medications such as tocilizumab, an IL-6 receptor blockade used for treating severe COVID-19 infections [19,31].

Other pro-inflammatory cytokines (IL-1β, IL-2, IL-8, IL-17, G-CSF, GMCSF, inducible protein-10 (IP-10), monocyte chemoattractant protein-1 (MCP-1), and TNFα) are significantly increased in severely ill patients [15,32,33]. Thus, elevated cytokines levels in the bloodstream may be used as a diagnostic/predictive factor in COVID-19 patients.

Interferons (INFs) are natural antiviral and immune-modulating agents that initially inhibit viral replication and enhance the immune response to improve clearance of the viral infection [34,35]. An impaired type I IFN response, featuring no IFN-β and low IFN-α production and activity, should be related to severe COVID-19 [36]. Paradoxically, delayed but exaggerated type I IFN responses are involved in hyper inflammation and contribute to the severe progression of COVID-19. These responses may be attributable to genetic and immunological factors [37].

### 2.2. C-Reactive Protein, Procalcitonin and Ferritin

As serum cytokines such as IL-6 and TNF-α are released, they further stimulate several downstream immune pathways, increasing the production of acute-phase reactants such as C-reactive protein (CRP) and procalcitonin (PCT). CRP is a non-specific acute-phase protein induced by IL-6 in the liver and a sensitive biomarker of inflammation, infection, and tissue damage [38].

CRP level is usually low in the bloodstream. Following acute inflammatory reactions, serum CRP increases significantly within 12–24 h, with a 20–72 h plateau and a subsequent return to baseline levels in 3–7 days [39,40,41]. As most CRP components are synthesized in the liver, liver failure would hinder the production of CRP [42]. COVID-19 patients with higher serum CRP are prone to evolve to severe disease states [43]. Among these COVID-19 patients, higher circulating CRP is associated with a higher rate of adverse events, such as venous thromboembolism events, acute kidney injury, and higher in-hospital mortality [44].

Procalcitonin (PCT), the precursor of calcitonin, is a glycoprotein consisting of a 116-amino acid, which is typically synthesized and released by thyroid parafollicular C cell [45,46]. Procalcitonin increases within four hours of response to infection or acute injury as an acute inflammatory marker, peaks at 6 h with a plateau at 8–24 h, and a return to baseline levels in 2–3 days [41]. Increased serum PCT level is associated with a high risk of bacterial infections and sepsis rather than viral infections, and PCT has previously been used to distinguish between bacterial and viral infections [47]. However, elevated PCT levels are correlated with the severity of SARS-CoV-2 infection [48,49,50]. Compared to moderate COVID-19 patients with abnormal image findings and clinical symptoms only, severe cases demonstrated a four to eight times higher procalcitonin level during severe or critical SARS-CoV-2 infection, respectively [50]. PCT levels appear to be useful as a disease severity predictor and may further imply concomitant bacterial infections. Co-infection rates were higher during severe SARS-CoV-2 infection and were accompanied by raised PCT levels [50]. For this reason, gradually increasing serum PCT levels may indicate a poorer prognosis.

Ferritin, an acute-phase protein, may interfere with iron metabolism [51]. Iron parameters, such as ferritin and transferrin, are not yet considered standard biomarkers for monitoring COVID-19 disease progression because the relationship between iron metabolism and COVID-19 remains unclear [52]. Some studies have claimed that elevated ferritin is a risk factor for COVID-19 severity [53,54,55].

## 3. Hematological Abnormality

SARS-CoV-2 infection alters the hematopoietic system and hemostasis, causing hematological abnormality and coagulopathy.

Lymphocytopenia, a hallmark of COVID-19, may be considered a crucial laboratory finding in terms of being a prognostic predictor. The degree of lymphopenia is correlated with the severity of the disease, while absolute lymphocyte counts (ALC) lower than 1000/mm^3^ indicate a poor prognosis [56]. The pathological mechanism of lymphopenia during severe COVID-19 disease states remains unclear, but the current evidence has led to multiple hypothesized mechanisms: (1) because SARS-CoV-2 is known to affect tissues (lung, heart, gastrointestinal tract) and induce ACE2 expression in the process, and because lymphocytes express ACE2 receptor on their surface [57], SARS-CoV-2 may directly infect lymphocytes and induce lysis or apoptosis; (2) the cytokine storm, a significantly increased cytokine response provoked by SARS-CoV-2 infection, attributes significant elevation of interleukins (primarily IL-6, IL-2, IL-7, granulocyte colony-stimulating factor, interferon-γ inducible protein 10, MCP-1, and MIP1-a) and raised tumor necrosis factor (TNF)-α, which leads to lymphocyte apoptosis [58,59,60]; (3) coexisting lactic acidosis may interfere with lymphocyte proliferation [61]; and, (4) increased cytokines may also contribute to lymphoid organ atrophy (including the spleen) and further hinder lymphocyte turnover [62].

From an adaptive immunity perspective, SARS-CoV-2 infection influences total lymphocyte count and balance. This hematological presentation, the result of an increase in circulating pro-inflammatory cytokines, is most impactful in patients with severe COVID-19 infection, who have shown significant reductions in the absolute number of CD4+, CD8+, B, and natural killer (NK) cells [63,64]. Additionally, decreased numbers of other mononuclear leukocytes, such as monocytes, eosinophils, and basophils, have also been documented [65], and may provide a strong predictive value for in-hospital mortality, organ injury, and severe respiratory injury and complications [66].

Eosinopenia seems to have a significant role in COVID-19 diagnosis and prognosis, as significantly reduced eosinophils were found in severe cases [67,68]. Eosinophils are primarily responsible for allergic reactions and anti-parasitic infections [69]. Previous animal studies have demonstrated an antiviral effect of eosinophils in various respiratory infections [70,71]. Persistent eosinopenia was associated with higher mortality, but an increased eosinophil count was associated with disease improvement [72,73]. Interestingly, asthmatic patients with higher eosinophil counts have lower hospitalization rates and are less likely to succumb to severe disease. Patients with pre-existing conditions associated with eosinophilia, such as asthma or allergic rhinitis, may be more protected from severe SARS-CoV-2 infection [67].

Neutrophilia in COVID-19 patients may be also be used as a predictive marker for disease severity. Following SARS-CoV-2 infection, the increased emergence of immature neutrophils in the blood has been directly correlated with the severity of COVID-19 [74,75]. The recruitment of neutrophils from the circulation into tissues occurs in most organs, especially in highly vascularized areas such as the lungs and kidneys [76]. This influx of neutrophils somehow damages neighboring vessels and parenchyma. Neutrophils are provoked to release neutrophil extracellular traps (NETs) upon facing threat signals. NETs immobilize and limit pathogens and facilitate their killing by antimicrobial agents. However, excessive formation or impaired removal would endanger the host [77]. The dysregulated NET formation may contribute to direct vascular injury and indirectly induce autoimmune vasculitis by forming autoantibodies [78]. Histopathological evidence suggests that NET-induced immune-thrombosis is associated with thrombotic events and organ damage in severe COVID-19 infections [79].

Neutrophil to lymphocyte ratio (NLR) and platelet to lymphocyte ratio (PLR) have been recognized as essential independent predictive factors for identifying at-risk COVID-19 patients [80,81]. Elevated NLR or PLR indicate that patient health is deteriorating and that they require increased oxygen, and that they are at higher risk of developing acute respiratory distress syndrome (ARDS) [82]. The unique hematological manifestations of SARS-CoV-2 infections may indicate the possible utility of a prognostic test base on hematological responses. Continuous assessment of lymphocyte count dynamics may provide valuable information for predicting patient prognoses. At the point of symptom onset, patients presenting lower lymphocyte percentages have been associated with increased disease severity over time [83].

### Hypercoagulation and Coagulopathy

Coagulation abnormalities and coagulopathy are associated with severe COVID-19 disease. Hyper-coagulation status, a unique manifestation of SARS-CoV-2 infection, results in thromboembolic events, such as deep vein thrombosis, pulmonary embolism, and microvascular thrombosis [84]. COVID-19 infection induces massive endothelial dysfunction and damage via direct virus invasion or indirect adaptive immunity mediated endothelial injury (either the NETs or complements) [84,85]. This immune response-triggered thrombosis causes widespread vascular damage, complement-induced thrombosis, systemic microangiopathy, and thromboembolism, that may ultimately lead to multi-organ failure [84,85,86]. Additionally, disseminated intravascular coagulopathy (DIC) with fulminant activation of coagulation, also reported in COVID-19 patients, has been shown to induce massive microvascular thrombosis and coagulation factor consumption [87]. Prolonged activated partial thromboplastin time, elevated D-dimer, and thrombocytopenia are all associated with the development of severe SARS-CoV-2 infection [88].

D-dimer is a soluble fibrin degradation product used as a marker for evaluating ongoing thrombotic and thrombolytic events such as pulmonary embolism [89]. D-dimer has been found in increased quantities among COVID-19 patients following the activation of coagulation and fibrinolysis [87]. As raised D-dimer has been associated with higher in-hospital mortality and increasing COVID-19 severity, it may be a reliable prognostic factor [90].

## 4. Organ Damage Indicator

### 4.1. Acute COVID–19 Cardiovascular Syndrome

A host of cardiovascular complications and involvements, called “acute COVID-19 cardiovascular syndrome”, occur during SARS-CoV-2 infection [91]. Acute coronary syndrome, myocardial injury, decompensated heart failure, stress-induced cardiomyopathy, pericardial effusion, viral myocarditis, or arrhythmia may be induced by various pathogenic routes following COVID-19 infection. Among patients with pre-existing cardiovascular comorbidities, cardiovascular diseases become more compromised or decompensated during COVID-19 infection. Systemic inflammatory response, thromboembolic events, and direct viral invasion compromise the cardiovascular system in COVID-19 patients, as do particular medication side-effects and hospital-acquired infections [91,92].

### 4.2. Cardiac Troponin

Myocardial injury is associated with poorer prognosis and outcome [91]. Cardiac troponins, consisting of troponin T, troponin I, and troponin C, are sensitive and specific for myocardial injury, and will appear elevated 4–10 h after acute myocardial ischemia [93]. They have been correlated with poor prognosis in cases involving pulmonary embolism or ischemic heart disease [92]. Cardiac complications have been more prevalent among COVID-19 cases compared to cases of SARS (severe acute respiratory syndrome). Epidemiological evidence indicates that 12% to 20% of hospitalized patients with COVID-19 have cardiac injuries, as implied by raised cardiac troponin levels [91,94]. For this reason, cardiac troponin may be useful as a potential prognostic marker to estimate patient outcome and mortality risk.

### 4.3. Brain Type NPs and N-Terminal Pro-BNP

Plasma natriuretic peptides (NPs), such as BNP (brain type NPs) and N-terminal pro-BNP (NT-proBNP), are commonly examined to evaluate acute heart failure (HF) patients [95]. Elevated NT-proBNP levels are associated with higher in-hospital mortality rates in COVID-19 cases [96] and provide more predictive value in combination with cardiac troponin [97]. However, although higher serum brain natriuretic peptide levels have been associated with cardiogenic pulmonary edema in COVID-19 patients with ARDS, some may have high levels of brain natriuretic peptide without significant ventricular dysfunction [98]. These cardiac biomarkers should be carefully considered when evaluating heart failure, as there are multiple mechanisms of cardiac injury. A point-of-care cardiac ultrasound should be considered to assess heart function to tailor treatment [98].

### 4.4. COVID 19-Associated Acute Renal Injury

In addition to the pulmonary manifestations associated with COVID-19, acute kidney injury (AKI) is a common complication during SARS-CoV-2 infection. Several pathogeneses injure renal tissues: local or systemic inflammatory responses and reactions, endothelial injury, coagulation-induced thromboembolic event, and renin–angiotensin system activation [99]. According to Kidney Disease Improving Global Outcomes (KDIGO), guidelines, which provides the consensus definition of AKI, previous studies have reported that nearly 30–50% of hospitalized patients with COVID-19 develop some form of AKI [100,101,102]. Near half of all patients in intensive care units (ICU) with acute kidney injury require renal replacement therapy [101,102], and in-hospital mortality is higher among patients with acute kidney injury [99]. The clinical manifestations of renal function deterioration range from mild proteinuria/hematuria to a drop in glomerular filtration rate (GFR), increased serum creatinine, or evolution to chronic kidney disease [99]. Notably, patients with pre-existing chronic kidney disease are vulnerable to severe clinical presentations and higher mortality [103]. For these reasons, deteriorated renal function or pre-existing renal disease during COVID-19 infection may indicate a poorer prognosis.

### 4.5. Markers for Other End-Organ Injury

Lactate dehydrogenase, an enzyme ubiquitously present in all human tissue, can be used as an indicator of gluconeogenesis and DNA metabolism [104]. Inflammatory responses and thromboembolic events hinder microcirculatory function and compromise oxygen delivery, increasing the likelihood of gluconeogenesis and DNA metabolism. Higher serum LDH might, therefore, be used to indicate COVID-19 disease severity and predict mortality risk [105,106,107].

Furthermore, higher serum lactate level is frequently considered an indicator of organ hypoperfusion or tissue hypoxia and is associated with more severe COVID-19 conditions. Higher serum lactate level is associated with poor prognosis and higher mortality [108,109].

### 4.6. Multisystem Inflammatory Syndrome

Multisystem inflammatory syndrome (MIS) is a rare but severe condition associated with post-COVID-19 infection in which various body parts become inflamed. MIS may involve the heart, lungs, kidneys, brain, skin, eyes, and/or the gastrointestinal organs. The presence of the symptoms in people <21 years old (or ≤19 years old per the World Health Organization definition is defined as MIS-children (MIS-C), and the presence of symptoms in people >21 years old is defined as MIS-adult (MIS-A). MIS-C is more common than MIS-A [8,110]. MIS shares similar clinical features with Kawasaki disease, an acute pediatric medium-vessel vasculitis. Symptoms, which include fever, elevated inflammatory markers, and multiple organ dysfunction, develop during or after SARS-CoV-2 infection [111].

Additional MIS-associated abnormalities include leukopenia/lymphopenia, elevated serum D-dimer, PCT, creatine kinase, and IL-6. Moreover, children with severe complications may have higher CRP, procalcitonin, or troponin levels and lower lymphocyte/platelet count [112,113]. The biomarkers listed in Table 2 may help establish the relationship between diagnostic criteria and clinical presentations [110,113].

## 5. Biomarkers in Different Clinical Courses

SARS-CoV-2 infection is characterized by triphasic clinical courses: initial manifestations, acute phase, and recovery. Some patients are asymptomatic during the first stage; however, symptomatic patients may present with cough, myalgias, headache, diarrhea, sore throat, or smell/taste abnormalities [115]. Mild lymphopenia was reported during this stage, but other hematological abnormalities were relatively rare.

Three-to-seven days after the initiation of symptoms, patients may enter the acute phase stage [115,116,117]. Some patients deteriorate to become severe cases during this stage, which variously includes respiratory failure, acute respiratory distress syndrome, and thromboembolic events. During this period, more severe hematological abnormalities, such as lymphopenia, neutrophilia, neutropenia, and thrombocytopenia develop, and immunological parameters such as IL-6, CRP, and PCT, increase in severe cases. Additionally, organ damage frequently occurs during this deteriorated stage. Elevated cardiac troponin, serum creatine, or D-dimer imply end-organ injury and poorer prognosis. This deterioration may occur as early as two days after admission, requiring intensive care, and some patients may later require intubation or ECMO. These parameters may improve after 15–16 days but if they do not, the persistent parameter abnormality is indicative of a poor prognosis [50,115,116,117]. Longitudinal and continuous monitoring of these parameters may help identify critical, at-risk patients and may therefore, provide impactful and timely intervention and improved outcomes.

The time to recover from COVID-19 infection is highly variable, accounting for age, pre-existing comorbidities, and illness severity. Most mild cases will fully recovery, but some long-term sequelae may persist in severe cases [118,119]. Different presentations of variable biomarkers are provided in Table 3.

## 6. Tailor to the Guidance of Treatment

Biomarkers are not only useful for prognostic evaluation but may be used for treatment guidance and response monitoring. These biomarkers may be useful for formulating targeted COVID-19 therapy. Serum IL-6 may help monitor treatment response and assess the efficacy of tocilizumab, an IL-6 receptor blockade, for treating severe COVID-19 cases [19,31]. COVID-19 patients with higher serum IL-6 levels may benefit more from IL-6 receptor antagonist therapy or the IL-6 receptor antagonist therapy combined with systemic corticosteroids [123,124]. Two classes of Food and Drug Administration (FDA.US)-approved IL-6 inhibitors: anti-IL-6 receptor monoclonal antibodies (mAbs) (e.g., sarilumab, tocilizumab) and anti-IL-6 mAbs (i.e., siltuximab) are now available for COVID-19 patients. However, these medications should be caution. Patients with systemic inflammations caused by non-SARS-CoV-2 viral infections, or other biologic immunomodulating drugs are not eligible for these medications [125].

Another pro-inflammatory cytokine or receptor inhibitor, the IL-1 receptor inhibitor, demonstrated some improved survival benefit for patients with COVID-19. Anakinra, a recombinant human interleukin-1 receptor (IL-1R) antagonist, has been approved to treat rheumatoid arthritis or other autoimmune disorder [126,127]. A meta-analysis has reported the administration of Anakinra in COVID-19 patients was safe and associated with reductions in both mortality and the requirement for mechanical ventilation [128]. A recent clinical trial, a double-blind, randomized controlled study, has successfully demonstrated the positive utility of using increased soluble urokinase plasminogen activator receptor(suPAR) to help select the best patients for Anakinra treatment. As the suPAR examination is currently widely used, one study also suggests that CRP, neutrophil-to-lymphocyte ratio (NLR), and ferritin may be used as alternative biomarkers [129]. However, because there is insufficient evidence regarding the use of IL1R antagonist for treating COVID-19 patients, COVID-19 treatment guidelines are not firm regarding the use of anakinra [130].

Reducing the level of serum pro-inflammatory cytokines may be used to treat COVID-19. Corticosteroids, therefore, may be used to decrease cytokine storm severity and improve patient mortality among patients infected with SARS-CoV-2 [127]. The RECOVERY trial showed that dexamethasone significantly reduced 28-day mortality in patients who needed respiratory support or extra oxygen [131]. In consideration of the adverse effect of corticosteroids, current guidance for COVID-19 treatment only recommends using corticosteroids in hospitalized patients who require oxygen or ventilation support [132]. On the other hand, interferon therapy for anti-viral therapy is not recommended currently because of the controversial effect on COVID-19 patients [133].

Changes in biomarkers may be used to indicate successful treatment and disease recovery. Following successful treatment, IL-2R, IL-6, TNF-α, and CRP levels decreased significantly among recovering COVID-19 patients. This was followed by reductions in IL-8, IL-10, and PCT. Further, CD4+ and CD8+ T lymphocytes normalized significantly, in patients who recovered, but only limited changes in B lymphocytes and natural killer cells were observed [134].

## 7. Conclusions

The clinical spectrum of SARS-CoV-2 infection is broad, ranging from asymptomatic, to mild, moderate, severe, and critical. To recognize case severity early, biomarkers may be used to provide additional and objective information, helping healthcare providers stratify COVID-19 patients accordingly. Here, we have reviewed: (1) the protective effect of eosinophilia during the initial presentation of the disease; (2) prognostic marker values associated with acute disease stages including various interleukins, CRP, PCT, as well as lymphopenia, neutrophilia, and other end-organ damage markers; and (3) recovery hallmarks of hematological neutralization and decreased cytokine levels. These biomarkers might also be leveraged for formulating treatment guidance, or individualized therapy based on the patient responses. We believe that more thorough review and clinical utilization of COVID-19 biomarkers could improve the quality of patient care and significantly improve the management of SARS-CoV-2 infections worldwide.

## Figures and Tables

**Table 1:** Classification of biomarkers that predicts the severity of COVID-19.

Host immune response	Cytokines Granulocyte macrophage-colony stimulating factor (GM-CSF) Interleukin 1β, (IL-1β,), IL-2, IL-6, IL-7, IL-8, IL-10, IL-17, TNF-α, interferon γ, IP-10, MCP-1 Inflammatory markers C-reactive Protein, Procalcitonin and Ferritin
Hematological Abnormality	Lymphopenia, Neutrophilia Decreased CD4+ CD8+ Decreased NK cell or B cell Neutrophil to Lymphocyte ratio Eosinopenia Thrombocytopenia
End-organ Injury	Cardiac troponin, Brain type NPs and N-terminal pro-BNP Serum creatinine Lactate dehydrogenase Serum lactate

**Table 2:** Diagnostic criteria for multisystem inflammatory syndrome in children (MIS-C) [110,114].

World Health Organization	Centers for Disease Control and Prevention (United States) *
Children and adolescents **0–19 years** of age with **fever > 3 days** AND **Two** of the following: ◆ Rash or bilateral non-purulent conjunctivitis or muco-cutaneous inflammation signs (oral, hands or feet). ◆ Hypotension or shock. ◆ Features of myocardial dysfunction, pericarditis, valvulitis, or coronary abnormalities (including ECHO findings or elevated Troponin/NT-proBNP), ◆ Evidence of coagulopathy (by PT, PTT, elevated d-Dimers). ◆ Acute gastrointestinal problems (diarrhea, vomiting, or abdominal pain). AND Elevated markers of inflammation such as ESR, C-reactive protein, or procalcitonin. AND No other obvious microbial cause of inflammation, including bacterial sepsis, staphylococcal, or streptococcal shock syndromes. AND Evidence of COVID-19 (RT-PCR, antigen test or serology positive), or likely contact with patients with COVID-19.	An individual **aged <21 years** presenting with fever >38.0 °C for ≥24 h Laboratory evidence of inflammation, **> 1 finding** ◆ An elevated C-reactive protein (CRP), erythrocyte sedimentation rate (ESR), fibrinogen, procalcitonin, d-dimer, ferritin, lactic acid dehydrogenase (LDH), or interleukin 6 (IL-6), elevated neutrophils, reduced lymphocytes, and low albumin Evidence of clinically severe illness requiring hospitalization, with **>2 organ involvement** ◆ Cardiac, renal, respiratory, hematologic, gastrointestinal, dermatologic, or neurological); AND No alternative plausible diagnoses. AND Positive for current or recent SARS-CoV-2 infection ◆ Confirmed by RT-PCR, serology, or antigen test; or exposure to a suspected or confirmed COVID-19 case within the *4 weeks prior to the onset of symptoms*.

* (1) Some individuals may fulfill full or partial criteria for Kawasaki disease but should be reported if they meet the case definition for MIS-C. (2) Consider MIS-C in any pediatric death with evidence of SARS-CoV-2 infection.

**Table 3:** The change of biomarkers referring severe cases in different clinical stage.

	Symptoms	Change of Biomarkers Referring to Severe Cases
Initial manifestations	Most patients are asymptomatic; Symptomatic patients may present with cough, myalgias, headache, diarrhea, sore throat, or smell/taste abnormalities [117].	Mild lymphopenia was reported during this stage, but other hematological abnormalities were relatively rare [120] Pre-existing comorbidities consisting of ↑ eosinophils may be a protective factor [67].
Acute phase	Mild to severe cases are admitted owing to desaturation, septic shock, or acute respiratory distress syndrome. Severe cases developed during this stage, complicated with respiratory failure, acute respiratory distress syndrome, thromboembolic events [117,118,119]	Immunologically Markedly ↑ interleukins (IL-1β, IL-2, IL-8, IL-17, G-CSF, GMCSF, inducible protein-10 (IP-10), monocyte chemoattractant protein-1 (MCP-1), and TNFα) ↓ or ↑ INFs * ↑ CRP and ↑ PCT, ↑ Ferritin Hematologically Markedly ↓ Lymphocyte ¶ ↓ CD4+ cells, CD8+ cells, B cells, and natural killers (NK) cells ↓ Eosinophils ↑ Neutrophils ↑ neutrophil to lymphocyte ratio (NLR), ↑ platelet and lymphocyte ratio (PLR) Hypercoagulation and coagulopathy ↑ D-Dimer End-Organ damage ↑ Cardiac Troponin, ↑ BNP, ↑ NT-proBNP, ↑ LDH, ↑ serum creatinine, ↑ serum lactate.
Recovery stage	Most mild cases will fully recovery. but some long-term sequelae might last in severe cases	Higher risk for neurological sequelae ↑ antibody response, ↑ plasma IL-6 levels, during acute phase [121]. Persistent biomarker abnormality The persist abnormality of the biomarkers during indicate the patients’ poor prognosis [50,115,116,117] ^§^ Post-COVID 19 multisystem inflammatory syndromes ^#^

* An impaired type I IFN response, featured by no IFN-β and low IFN-α production and activity, should be related to severe COVID-19 [36]. Delayed but exaggerated type I IFN responses, however, contribute to the severe progression of COVID-19 [37]; ¶ The degree of lymphopenia is correlated with disease severity, while absolute lymphocyte counts (ALC) lower than 1000/mm^3^ indicate a poor prognosis [56]; ^§^ Hematological abnormality generally recovered on day 15–16 after admission [122]; ^#^ Biomarkers and symptoms related to multisystem inflammatory syndromes are listed in Table 2.

## Data Availability

Not applicable.
